# Tannin in Ophthalmic Diseases

**Published:** 1851-08

**Authors:** 


*Tannin in Ophthalmic Diseases.*—M. Hairion of Louvain has a very high opinion of Tannin in eye-diseases. The following remarks by him are quoted from the *Journal de Médecine et de Chirurgie Pratiques* for October 1850.

Tannin has, probably, been only used in catarrhal ophthalmia, in the proportion of one part to 120 of water; but the want of efficacy of this solution has made it fall into almost complete disuse. For more than five months, I have employed tannin in the form of pommade, in thick mucilage, in fine powder, and especially in concentrated solution (one part to three of water). The affections in which it has proved most successful have been acute and chronic blennorrhœa, œdematous swelling of the conjunctivæ, vegetating granulations, vascular and ulcerative affections of the cornea, and especially pannus, which has been cured, in some cases, with astonishing rapidity.

Tannin has been justly styled the styptic *par excellencc;* its local mechanico-chemical action renders it highly useful in ophthalmology, whether to retard muco-purulent effusion of the conjunctiva, to combat the relaxation of that membrane, to obtain the obliteration of cellulo-vascular productions, or to diminish dilated or newly-formed vessels; or, by producing coagulation of the plastic fluids, to accelerate the cicatrisation of ulcers of the cornea, strengthen its softened tissue, and prevent its projection or laceration.

This remedy is free from the objections which may be urged against other astringents. Its application is not painful, it produces no severe reaction, and leaves no caustic marks, nor indelible incrustations.

Tannin has been employed in the circumstances indicated above, frequently with signal success, sometimes without advantage, never with injury.—*Lon. Jour. of Med., March,* 1851.

